# Simple Synthesis of High Specific Surface Carbon Nitride for Adsorption-Enhanced Photocatalytic Performance

**DOI:** 10.1186/s11671-018-2654-7

**Published:** 2018-08-22

**Authors:** Jie Wang, Meisheng Li, Ming Qian, Shouyong Zhou, Ailian Xue, Lili Zhang, Yijiang Zhao, Weihong Xing

**Affiliations:** 1 Jiangsu Engineering Laboratory for Environmental Functional Materials, Jiangsu Key Laboratory for Chemistry of Low-Dimensional Materials, School of Chemistry and Chemical Engineering, Huaiyin Normal University, No.111 West Changjiang Road, Huaian, 223300 Jiangsu Province People’s Republic of China; 2 College of Chemical Engineering, Nanjing Tech University, No.5 Xinmofan Road, Nanjing, 210009 Jiangsu Province People’s Republic of China

**Keywords:** Carbon nitride materials, Melamine, Nanoparticles, Sintering

## Abstract

**Abstract:**

TMC-incorporated carbon nitride (CN) with hexagonal and quadrangle honeycomb-like structure and having periodic lattice defects linked by –CONH– bond was synthesized through combining the high calcination with the chemical condensation of melamine and 1,3,5-benzenetricarbonyl trichloride. The obtained CN has a tri-s-triazine ring and benzene ring skeleton, which makes it have excellent mechanical and thermal stability. The BET specific surface area was enhanced to about 125.6 m^2^/g, and the mean pore size is about 3.43 nm. This CN exhibited an excellent adsorption-enhanced photocatalytic performance.

**Graphical abstract:**

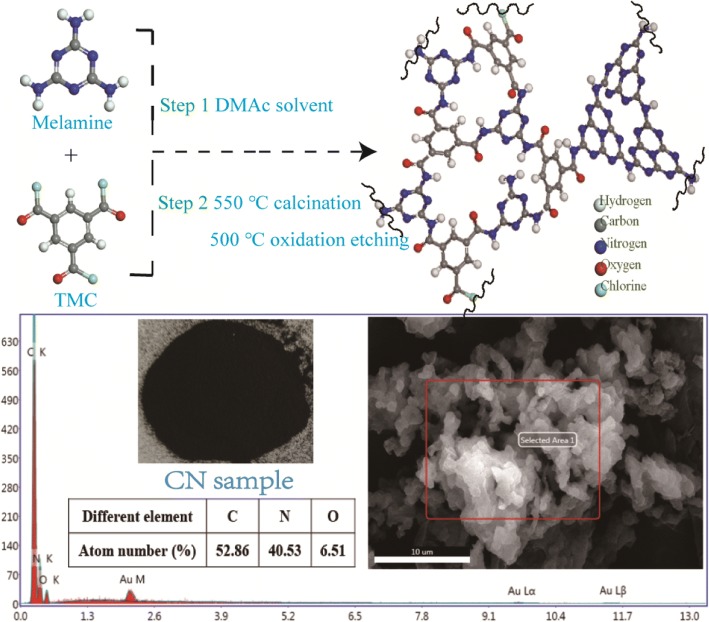

**Electronic supplementary material:**

The online version of this article (10.1186/s11671-018-2654-7) contains supplementary material, which is available to authorized users.

## Background

Graphite carbon nitride (g-C_3_N_4_), possessing a two-dimensional (2D) nanosheet structure like graphene, has attracted much attention recently. The basic skeleton structure of g-C_3_N_4_ consists of tri-s-triazine units connected with tertiary amino groups, which owns regularly distributed triangular water-selective permeation nanopores throughout the entire laminar structure. Moreover, the spacers between the g-C_3_N_4_ nanosheets, which interact with each other through weak van der Waals forces, also provide nanochannels for water transport while bigger molecules are retained [1]. Due to this unique nanosheet structure, g-C_3_N_4_ exhibited many useful properties with applications in many fields, such as membrane separation materials [2, 3], photocatalysis [4–6], and electronic devices [7–9]. Up to now, the main method to prepare g-C_3_N_4_ was high-temperature calcination. However, the specific BET surfaces of these g-C_3_N_4_ materials were only 5–35 m^2^/g [2, 4, 10], which seriously limits the displaying of their unique properties in applications [4]. Then, the enhancement of specific BET surface for g-C_3_N_4_ was very important.

Inspired by the structures of covalent organic framework materials (COFs) [11–16] and their preparation methods [17], we try to combine the high calcination with the chemical condensation to prepare a new kind of porous TMC-incorporated carbon nitride (CN) linked by –CONH– bond and having periodic lattice defects as well [18]. This CN was synthesized by the condensation of melamine and 1,3,5-benzenetricarbonyl trichloride through the reaction of –NH_2_ and –COCl. Besides, due to the further calcination and thermal oxidation “etching” using melamine as a precursor, this CN could possess large numbers of –NH and –NH_2_ groups in the lattice structure, which could endow the CN with excellent performances.

## Methods/Experimental

### Materials

Melamine (99%), 1,3,5-benzenetricarbonyl trichloride (TMC, 98%), *N*-*N*-dimethylacetamide (DMAc, ≥ 99.8%), *N*,*N*-dimethylformamide (DMF, 99.5%), and triethyl phosphate (TEP, ≥ 99.5%) were purchased from Aladdin Chemistry Co., Ltd. (China). Ethanol (≥ 99.7 wt.%) and acetic acid (≥ 99.5 wt.%) were purchased from Sinopharm Chemical Reagent Co., Ltd. (Shanghai, China). Purified water was purchased from Hangzhou Wahaha Group Co., Ltd. (Hangzhou, China). All the materials and reagents were of analytical grade and used without further purification.

### Synthesis of CN Material

**Scheme 1:**
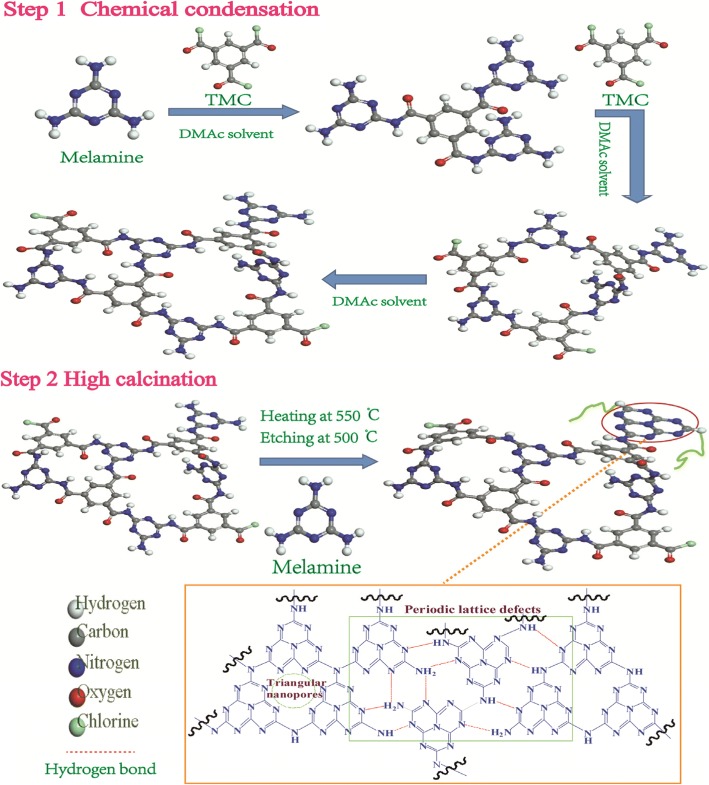
Proposed reaction mechanism of melamine and 1,3,5-benzenetricarbonyl trichloride for the synthesis of the TMC-incorporated carbon nitride

The synthesis procedure of CN material is illustrated in Scheme 1. At first, a white suspension was obtained by dissolving melamine (5 g) and TMC (1.75 g) in DMAc (93.25 g) with the stirring at 80 °C for 3 h under oil bath reflow (Additional file 1: Table S1–S3 and Figure S1–S3). Then, the suspension was transferred into roller ball mill to mill 12 h, using zirconia porcelain balls as the ball milling media. After that, the suspension was followed by ultrasonic treatment for 3 h. Secondly, the suspension was filtrated and washed with ethanol and deionized water for three times, respectively. In the end, the resulted white precipitate was dried at 80 °C in vacuum drying oven for about 24 h.

The above white powder was heated in two steps at 550 °C and 500 °C, respectively. Specifically, the procedure consisted in an initial heating from room temperature (25 °C) to 500 °C, which required 150 min, holding for 120 min. Then, the temperature was increased to 550 °C in 10 min and holding for 240 min. After that, the temperature was allowed to drop back to 25 °C, which took 100 min. The obtained bulk CN was milled into powder. Finally, the bulk CN was transferred into an open ceramic boat and heated to 500 °C in air for 4 h with a ramp rate of 10 °C/min. The yield is about 25%.

### Characterizations

The size and morphology were analyzed by scanning electron microscopy (SEM, QUANTA FEG450) and transmission electron microscopy (TEM, Tecnai G2 F30 S-TWIN). The crystalline structure was confirmed by X-ray diffraction (XRD, Switzerland ARL/X, TRA). Fourier transform infrared spectrometry (FT-IR, Nicolet is50) was used to study the chemical structure. Thermal stability was inspected using thermal gravimetric analysis and differential scanning calorimetry (TG-DSC, STA449F3). X-ray photoelectron spectroscopy (XPS, Thermo ESCALAB 250) signals were recorded with a monochromatic Al Ka source and a change neutralizer (hv = 1486.6 eV). Conventional method for measurement of the BET specific surface and average pore sizes were carried out by a B40-SA3100PLUS specific surface area and void meter (BECKMAN COULTER). The particle size distribution was analyzed by ZETSIZER NANO (ZSE, Malvern Instruments Ltd., UK).

Photodecomposition of 20 mg/L and 10 mg/L methyl orange (MO) were chosen as the model system to investigate the photoactivity of products using XPA photochemical reactor under visible-light irradiation of a 500-W Xe lamp with a 420-nm cutoff filter. A certain amount known concentration of H_2_O_2_ was then added into the reactor after 30 min dark adsorption, and the lamp was turned on to run 120 min continuously. Five milliliters of samples were withdrawn at regular intervals (20 min) with centrifugation to separate solids for measure. Furthermore, the CN suspension was filtered by a filter membrane and washing three times by deionized water and absolute ethanol, respectively. Then, the CN was dried in an oven at 80 °C for 3 h. The obtained CN was used to degrade MO again according to the above steps. The recycling experiments were carried out three times.

In order to prove the structure of the synthesized CN material, the XRD patterns of the CN molecular structure was simulated by Material Studio software (MS 2017, ver. 17.1.). The lattice models (e.g., cell parameters, orientation standard, atomic positions, and total energies) were then fully optimized using MS Forcite and Reflex modules method (Additional file 1: Figure S9).

## Results and Discussion

### Chemical Structure of CN Material

**Fig. 1:**
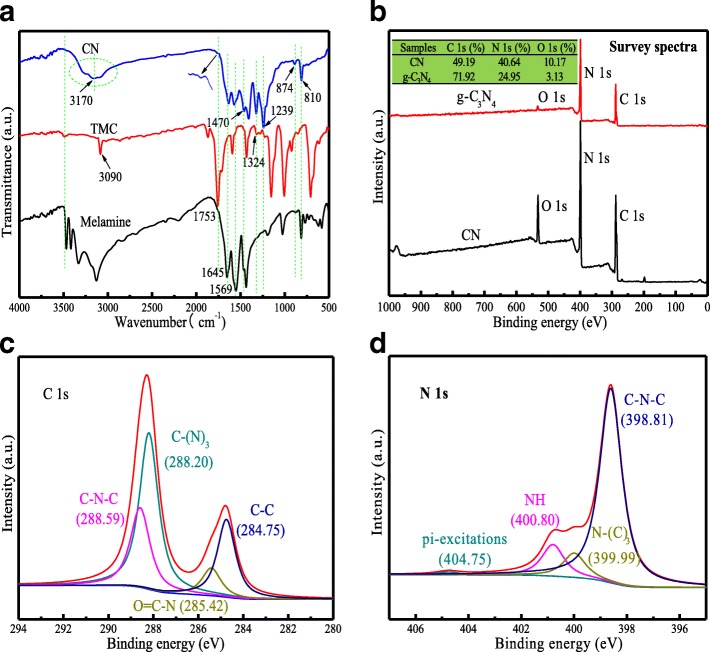
**a** FT-IR spectra, **b** XPS survey spectrum, the high-resolution scan of **c** C1s and **d** N1s of CN material

Figure 1a depicts the chemical structure of CN. The sharp peak at 810 cm^−1^ was assigned to the s-triazine ring mode. The characteristic peaks appearing at 1239 cm^−1^, 1324 cm^−1^, 1470 cm^−1^, 1569 cm^−1^, and 1645 cm^−1^ were related to the C–NH–C and N–(C)_3_ stretching vibration modes [19]. The peak at 1753 cm^− 1^ was belonged to –CONH– vibration, which was often called amide I band. A broad absorption around 3170 cm^−1^ was attributable to stretching modes of primary and secondary amines at the defect sites and their intermolecular hydrogen bonding interactions [2].

**Fig. 2:**
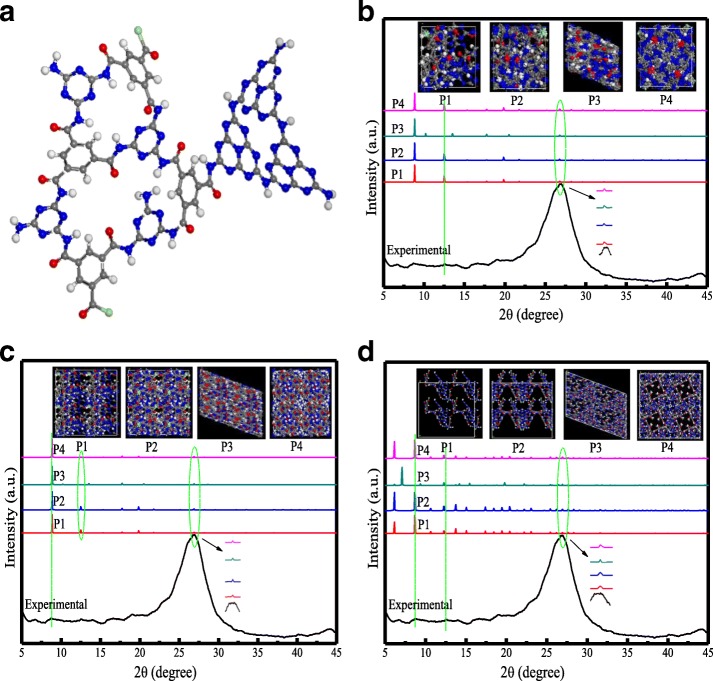
**a** Energy-optimized structural monomer representation and **b**–**d** the comparison of XRD patterns for CN material

XPS was further employed to investigate the surface chemical composition and chemical states of the CN. As shown in Fig. 1b, the C/N ratio of CN was about 1:1, which was far beyond the traditional carbon-rich g-C_3_N_4_ [2, 20–22] (C/N ratio, about 3:1). Thus, this prepared material is the type of nitrogen-rich g-C_3_N_4_ [22] and we marked it as CN, the same phenomenon was obtained from the EDX element analysis in Additional file 1: Figure S5, 6, wherein only carbon, nitrogen, and oxygen species were detected (Fig. 1b). Besides, the O1s peak at 531.9 eV was likely due to the surface adsorbed H_2_O or hydroxyl group. As can be seen, the four same peaks in Fig. 1c at 288.59 eV, 288.20 eV, 285.42 eV, and 284.75 eV were ascribed to C–N–C, C–(N)_3_, O=C–N, and C–C groups, respectively. From the high-resolution scan of N1s (Fig. 1d), four typical peaks are ascribed to the nitrogen in C–N–C (398.81 eV), N–(C)_3_ (399.99 eV), N–H (400.80 eV), and pi-excitations groups (404.75 eV) [3], respectively. Furthermore, from the -resolution scan of O1s (Additional file 1: Figure S7), the two peaks at 533.12 eV and 531.82 eV were ascribed to C–O and C=O groups, respectively. The C=O was mainly ascribed to the C=O–N and C=O–C groups in the TMC and the generated –CONH–. The C–O peak may be due to the surface oxygen cavity or hydroxyl group. In all, from above analysis results, the possible detailed monomer structure is shown in Fig. 2a, and the CN material was prepared in a brief process successfully.

The XRD characterization in Additional file 1: Figure S4a-c was indicated that the crystal structure of CNs were not destroyed by different solvents, temperature, and addition amount of TMC. From Fig. 2b–d, we know that the strong peak located at 26.96° corresponded to the stacking of conjugated aromatic planes (002 planes) and the relatively weak peak at 12.45° was assigned to in-plane structural packing motif of tri-s-triazine units (100 planes), respectively. When different space groups were used to build CN to obtain XRD patterns, typical characteristic peaks of CN were perfectly displayed in the simulated XRD patterns. Thus, the CN structure predicted in this work is reasonable.

### Physical Structure of CN Material

**Fig. 3:**
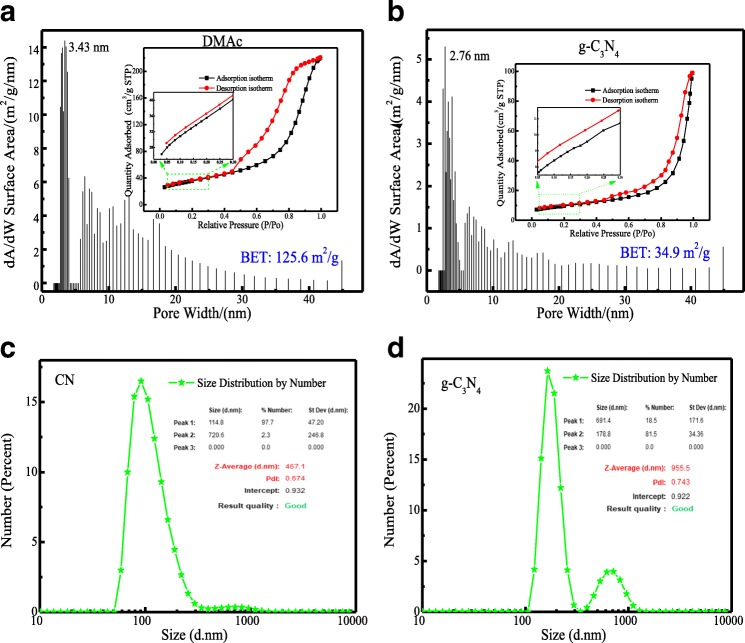
Pore size distribution of **a** CN and **b** g-C_3_N_4_. Inset: N_2_ adsorption and desorption isotherm measured at − 195.671 °C and the particle size of **c** CN and **d** g-C_3_N_4_

The permanent porosity of CN was demonstrated by N_2_ adsorption-desorption analysis at − 195.671 °C (Fig. 3a). The isotherm shows a sharp uptake below P/P0 = 1.0, and the Brunauer-Emmett-Teller (BET) surface area was calculated to be 125.6 m^2^/g. Compared with traditional g-C_3_N_4_ materials (5–35 m^2^/g [2, 20, 21], Fig. 3b and Additional file 1: Table S4), the BET surface of this new CN was enhanced greatly. From Scheme 1, through the reactions between –NH_2_ and –COCl in melamine and TMC, melamine could graft to TMC strongly and regularly, thereby dispersing uniformly in DMAc solution. Then, this process may provide more grafting sites for the subsequent calcination of melamine and improved the BET specific surface area greatly. As shown in Fig. 3c, d, the average particle size of CN was 467.1 nm, which was smaller than the 955.5 nm of g-C_3_N_4_. It is also proved that the CN material owned a large BET surface.

**Fig. 4:**
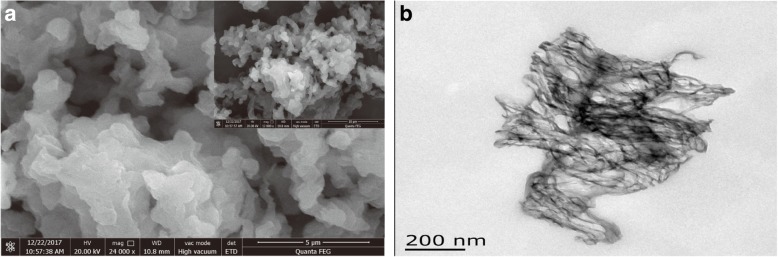
**a** SEM and **b** TEM images of CN material

It was seen that the CN showed a uniformly distributed and loose block morphology from Fig. 4a. From the Additional file 1: Figure S8, the different CN layer structures were arranged orderly, and there was no obvious agglomeration. Besides, there were many pores in this CN due to the introduction of TMC. Moreover, as clearly observed in TEM (Fig. 4b), the CN owned many wrinkles and grooves. These are just the main reasons for that the CN has a high BET surface.

**Fig. 5:**
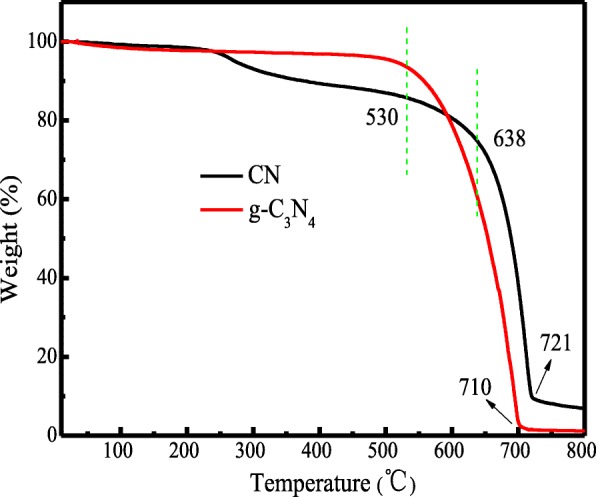
TGA curves of CN material at 80 °C under the solvent of DMAc

The results of TGA curves are shown in Fig. 5. From Fig. 5, as the most stable allotrope of carbon nitride, the prepared CN material and g-C_3_N_4_ showed a unique temperature resistance up to 721 °C and 710 °C, respectively. From the result, it can be concluded that the CN is thermally stable in oxygen condition under 530 °C.

### Adsorption-Enhanced Photoactivity of CN Material

**Fig. 6:**
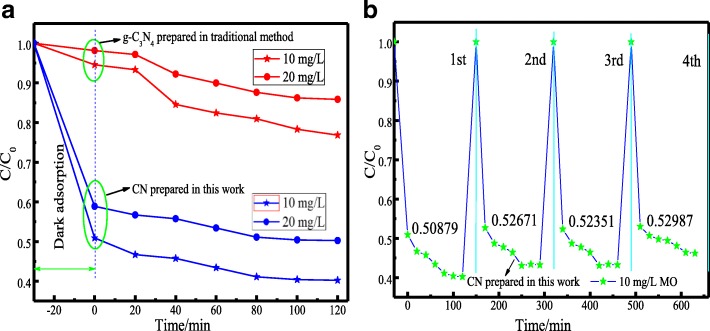
**a** Comparison of photoactivity between CN and g-C_3_N_4_ and **b** recycling degradation experiments of CN

The photoactivity of 20 mg/L and 10 mg/L methyl orange (MO) was investigated by XPA photochemical reactor, which is shown in Fig. 6 (the C_0_ is the absorbance of MO in initial concentration (20 mg/L and 10 mg/L), the C is the absorbance of MO at different time) [20].

From Fig. 6a, this new CN had a high MO adsorption performance due to the large BET surface. After 120 min, the photoactivity was reached 60% far beyond the photoactivity of g-C_3_N_4_ [2], which was about 20%. The CN exhibited an excellent adsorption-enhanced photoactivity, even after 4 cycles under identical conditions (Fig. 6b). As we know, photo induced electron-hole pairs were generated on the surface of CN after absorption visible-light photons which were equal or higher than its band gap. During the photocatalytic process, while the photoelectron of CN reacted with oxygen to generate ⋅O_2_^−^ and ⋅OH. Subsequently, ⋅O_2_^−^ and ⋅OH combined with MO to further decompose into CO_2_ and H_2_O. However, the periodic lattice defects in the CN and g-C_3_N_4_ would capture photo-generated electrons, thereby reducing the photocatalytic efficiency [20]. Thus, the photoactivities of single CN and g-C_3_N_4_ materials were not high, which should be combined with other materials.

## Conclusions

A new –CONH– bond linked CN material with hexagonal and quadrangle honeycomb-like structure was constructed by combining the high calcination with the chemical condensation of melamine and 1,3,5-benzenetricarbonyl trichloride. This material has a tri-s-triazine ring and benzene ring skeleton structure. The BET specific surface area of CN was about 125.6 m^2^/g, and the mean pore size is about 3.43 nm. This CN exhibited an excellent adsorption-enhanced photocatalytic performance.

## Additional file

Additional file 1: **Table S1**. BET surface areas and average pore sizes of CNs prepared at 80 °C with the addition amount of 1.0 g TMC under different solvents. **Table S2.** BET surface areas and average pore sizes of CNs prepared at different temperature with the addition amount of 1.0 g TMC under the solvent of DMAc. **Table S3.** BET surface areas and average pore sizes of CNs prepared with different addition amount of TMC under the solvent of DMAc at 80 °C. **Figure S1.** Pore size distribution of CNs at 80 °C with the addition amount of 1.0 g TMC under different solvents. (a) H_2_O, (b) CH_3_COOH, (c) DMAc, (d) TEP, (e) DMF. Inset: N_2_ adsorption and desorption isotherm for CNs measured at − 195.671 °C. **Figure S2.** Pore size distribution of CNs at different temperature with the addition amount of 1.0 g TMC under the solvent of DMAc. (a) 50 °C, (b) 60 °C, (c) 70 °C, (d) 90 °C, (e) 100 °C. Inset: N_2_ adsorption and desorption isotherm for CNs measured at − 195.671 °C. **Figure S3.** Pore size distribution of CNs with different addition amount of TMC under the solvent of DMAc at 80 °C. (a) 1.5 g, (b) 2.0 g, (c) 2.5 g. Inset: N_2_ adsorption and desorption isotherm for CNs measured at − 195.671 °C. **Table S4.** The comparison of BET surface areas of CNs. **Figure S4.** XRD of different CNs (a) different solvents, (b) different temperature and (c) different addition amount of TMC. **Figure S5.** EDX spectra of (a) H_2_O, (b) CH_3_COOH, (c) DMAc, (d) TEP, (e) DMF. **Figure S6.** The element analysis of EDX. **Figure S7.** The high resolution scan of O1s of CN material. **Figure S8.** SEM images of CNs under different solvents. (a) H_2_O, (b) CH_3_COOH, (c) TEP, (d) DMF. **Figure S9.** Flow chart of simulation calculation of CN material. (DOC 8323 kb)
