# Supplementary material for: Simple Synthesis of High Specific Surface Carbon Nitride for Adsorption-Enhanced Photocatalytic Performance
Source: Nanoscale Res Lett. 2018 Aug 22;13:248. doi: 10.1186/s11671-018-2654-7 (PMC6104408; doi:10.1186/s11671-018-2654-7)
Supplement: Supplementary file 1 — Table S1. BET surface areas and average pore sizes of CNs prepared at 80 °C with the addition amount of 1.0 g TMC under different solvents. Table S2. BET surface areas and average pore sizes of CNs prepared at different temperature with the addition amount of 1.0 g TMC under the solvent of DMAc. Table S3. BET surface areas and average pore sizes of CNs prepared with different addition amount of TMC under the solvent of DMAc at 80 °C. Figure S1. Pore size distribution of CNs at 80 °C with the addition amount of 1.0 g TMC under different solvents. (a) H2O, (b) CH3COOH, (c) DMAc, (d) TEP, (e) DMF. Inset: N2 adsorption and desorption isotherm for CNs measured at − 195.671 °C. Figure S2. Pore size distribution of CNs at different temperature with the addition amount of 1.0 g TMC under the solvent of DMAc. (a) 50 °C, (b) 60 °C, (c) 70 °C, (d) 90 °C, (e) 100 °C. Inset: N2 adsorption and desorption isotherm for CNs measured at − 195.671 °C. Figure S3. Pore size distribution of CNs with different addition amount of TMC under the solvent of DMAc at 80 °C. (a) 1.5 g, (b) 2.0 g, (c) 2.5 g. Inset: N2 adsorption and desorption isotherm for CNs measured at − 195.671 °C. Table S4. The comparison of BET surface areas of CNs. Figure S4. XRD of different CNs (a) different solvents, (b) different temperature and (c) different addition amount of TMC. Figure S5. EDX spectra of (a) H2O, (b) CH3COOH, (c) DMAc, (d) TEP, (e) DMF. Figure S6. The element analysis of EDX. Figure S7. The high resolution scan of O1s of CN material. Figure S8. SEM images of CNs under different solvents. (a) H2O, (b) CH3COOH, (c) TEP, (d) DMF. Figure S9. Flow chart of simulation calculation of CN material. (DOC 8323 kb) [file 11671_2018_2654_MOESM1_ESM.doc]

**Supporting information for**

**Simple synthesis of high specific surface carbon nitride for adsorption-enhanced photocatalytic performance**

**Jie Wang^a,b^, Meisheng Li^a,*^, Ming Qian^a^, Shouyong Zhou^a^, Ailian Xue^a^, Lili Zhang^a^, Yijiang Zhao^a,*^, Weihong Xing^b^**

*^a^ School of Chemistry and Chemical Engineering, Huaiyin Normal University,* *Jiangsu Engineering Laboratory for Environmental Functional Materials, Jiangsu Key Laboratory for Chemistry of Low-Dimensional Materials, No.111 West Changjiang Road, Huaian 223300, Jiangsu Province, PR China.*

*^b^ College of Chemical Engineering, Nanjing Tech University, No.5 Xinmofan Road, Nanjing 210009, Jiangsu Province, PR China.*

**Corresponding author: E-mail: lms1108@hytc.edu.cn (Meisheng Li)*

*E-mail: c*[*yjzhao@126.com*](mailto:yjzhao@126.com) *(Yijiang Zhao)*

**1.1 Tables and Figures**

**Table S1.** BET surface areas and average pore sizes of CNs prepared at 80 ℃ with the addition amount of 1.0 g TMC under different solvents.

| Samples (Solvent) | H_2_O | CH_3_COOH | DMAc | TEP | DMF |
| --- | --- | --- | --- | --- | --- |
| BET Surface Area/(m²/g) | 45.8815 ± 0.2673 | 34.5280 ± 0.1692 | 91.1222 ± 0.2722 | 63.9615 ± 0.1229 | 51.3874 ± 0.2131 |
| Adsorption and Desorption average pore diameter (4V/A by BET)/(nm) | 17.56279 | 15.29768 | 12.85254 | 19.15910 | 20.07324 |
|  | 20.04885 | 17.19451 | 13.15430 | 20.85554 | 22.03767 |

**Table S2.** BET surface areas and average pore sizes of CNs prepared at different temperature with the addition amount of 1.0 g TMC under the solvent of DMAc.

| Temperature (DMAc)/( ℃) | 50 | 60 | 70 | 80 | 90 | 100 |
| --- | --- | --- | --- | --- | --- | --- |
| BET Surface Area/(m²/g) | 28.4197 ± 0.0858 | 30.4814 ± 0.1579 | 72.0166 ± 0.2249 | 91.1222 ± 0.2722 | 34.1945 ± 0.1086 | 56.2157 ± 0.1278 |
| Adsorption and Desorption average pore diameter (4V/A by BET)/(nm) | 15.45385 | 19.02616 | 14.42018 | 12.85254 | 17.42998 | 15.68535 |
|  | 15.84995 | 19.65808 | 14.69486 | 13.15430 | 17.93999 | 16.03528 |

**Table S3.** BET surface areas and average pore sizes of CNs prepared with different addition amount of TMC under the solvent of DMAc at 80 ℃.

| TMC/(g) | 1.0 | 1.5 | 1.75 | 2.0 | 2.5 |
| --- | --- | --- | --- | --- | --- |
| BET Surface Area/(m²/g) | 91.1222 ± 0.2722 | 111.4903 ± 0.1508 | 125.5515 ± 0.3381 | 104.6274 ± 0.1857 | 77.7387 ± 0.2606 |
| Adsorption and Desorption average pore diameter (4V/A by BET)/(nm) | 12.85254 | 12.15143 | 10.84324 | 11.75470 | 11.85570 |
|  | 13.15430 | 12.23830 | 10.88398 | 11.82960 | 12.03471 |

**Fig. S1**. Pore size distribution of CNs at 80 ℃with the addition amount of 1.0 g TMC under different solvents. (a) H_2_O, (b) CH_3_COOH, (c) DMAc, (d) TEP, (e) DMF. Inset: N_2_ adsorption and desorption isotherm for CNs measured at -195.671 ℃.

**Fig. S2**. Pore size distribution of CNs at different temperature with the addition amount of 1.0 g TMC under the solvent of DMAc. (a) 50 ℃, (b) 60 ℃, (c) 70 ℃, (d) 90 ℃, (e) 100 ℃. Inset: N_2_ adsorption and desorption isotherm for CNs measured at -195.671 ℃.

**Fig. S3**. Pore size distribution of CNs with different addition amount of TMC under the solvent of DMAc at 80 ℃. (a) 1.5 g, (b) 2.0 g, (c) 2.5 g. Inset: N_2_ adsorption and desorption isotherm for CNs measured at -195.671 ℃.

**Table S4.** The comparison of BET surface areas of CNs.

| Samples | BCN [1] | CNNS [1] | g-C_3_N_4_ [2] | ag-C_3_N_4_ [2] | Bulk g-C_3_N_4_ [3] | g-C_3_N_4_ [3] |
| --- | --- | --- | --- | --- | --- | --- |
| BET Surface Area/(m²/g) | 4.7 | 31.1 | 12.7 | 26.2 | 6-10 | 34.9 |

Note: the reference [3] is my previous work.

**Fig. S4**. XRD of different CNs (a) different solvents, (b) different temperature and (c) different addition amount of TMC.


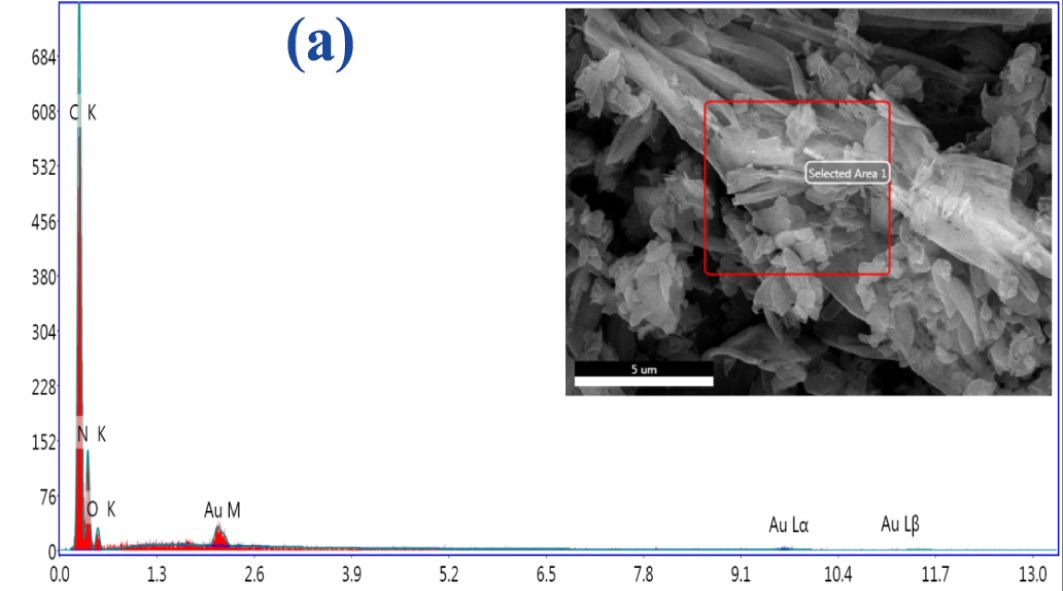


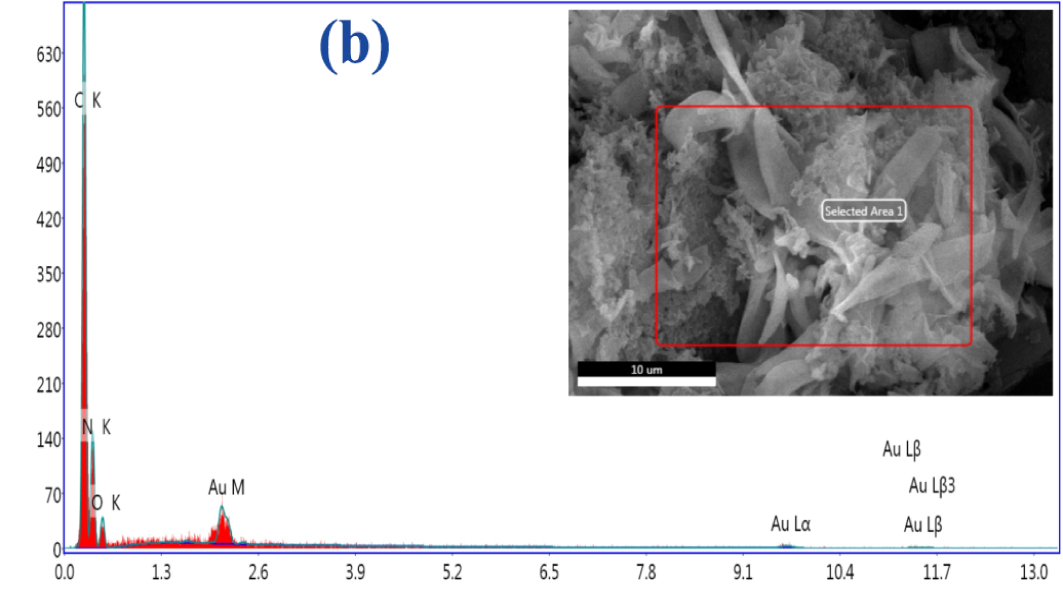


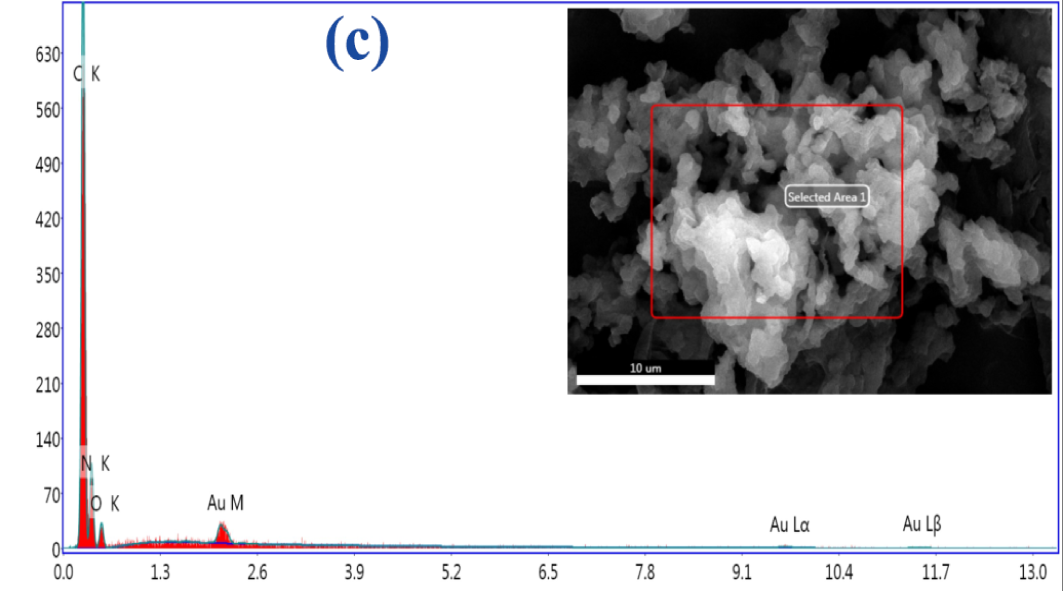


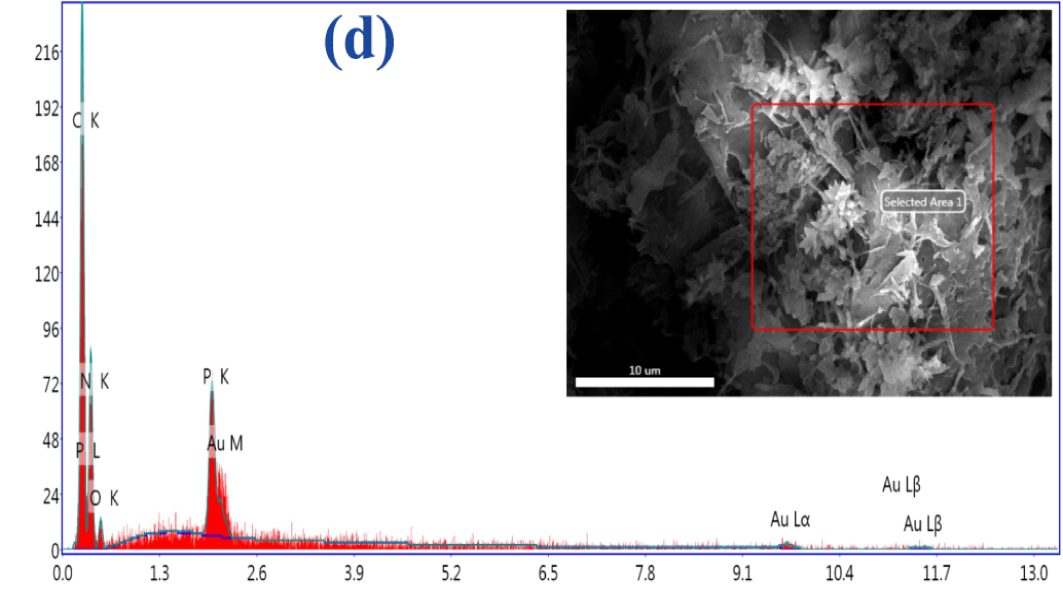


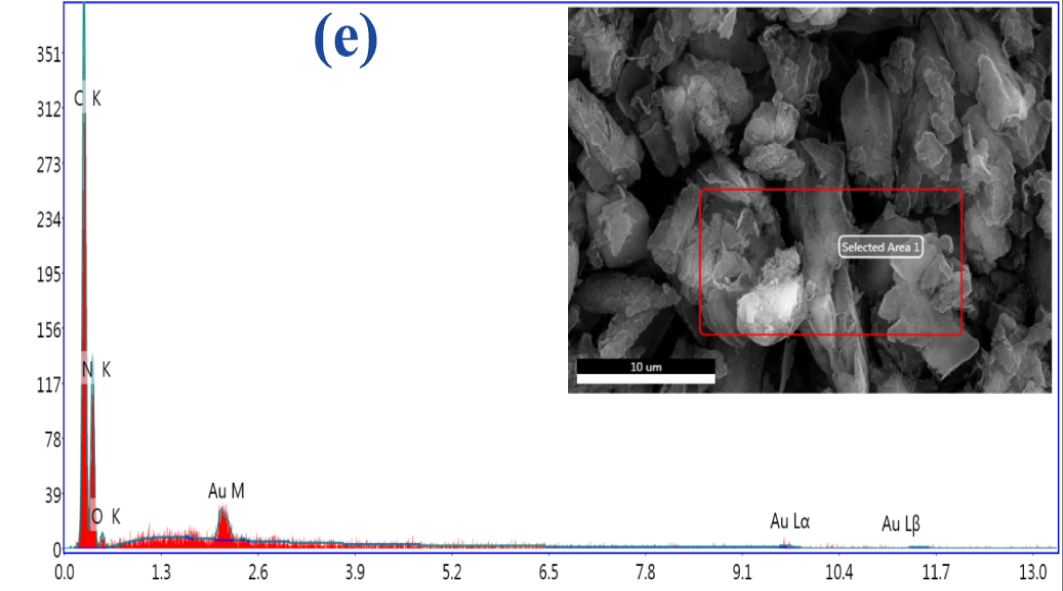


**Fig. S5**. EDX spectra of (a) H_2_O, (b) CH_3_COOH, (c) DMAc, (d) TEP, (e) DMF.

**Fig. S6**. The element analysis of EDX.

**Fig. S7.** The high resolution scan of O1s of CN material.


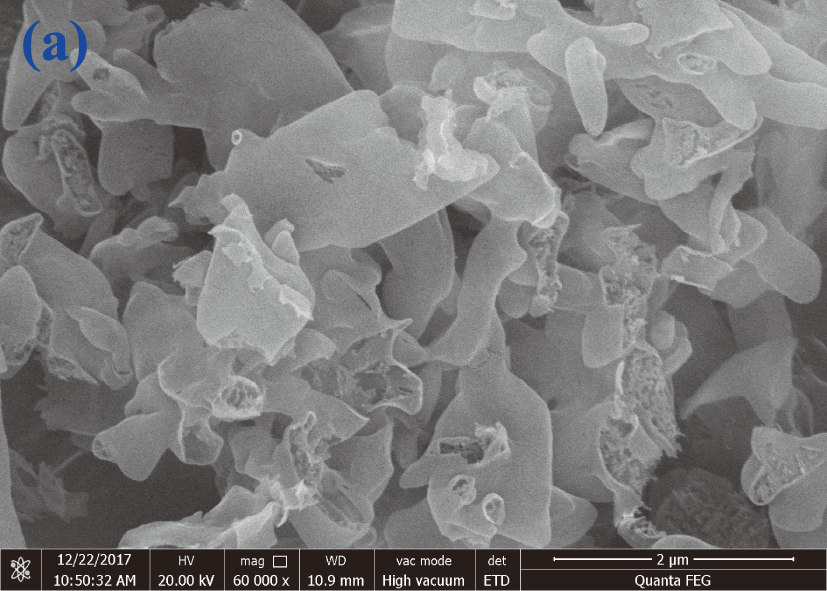

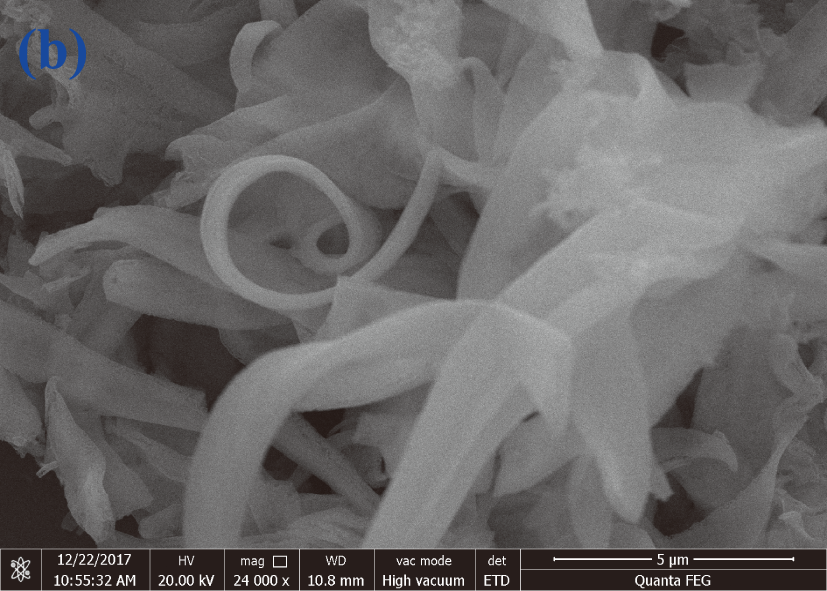


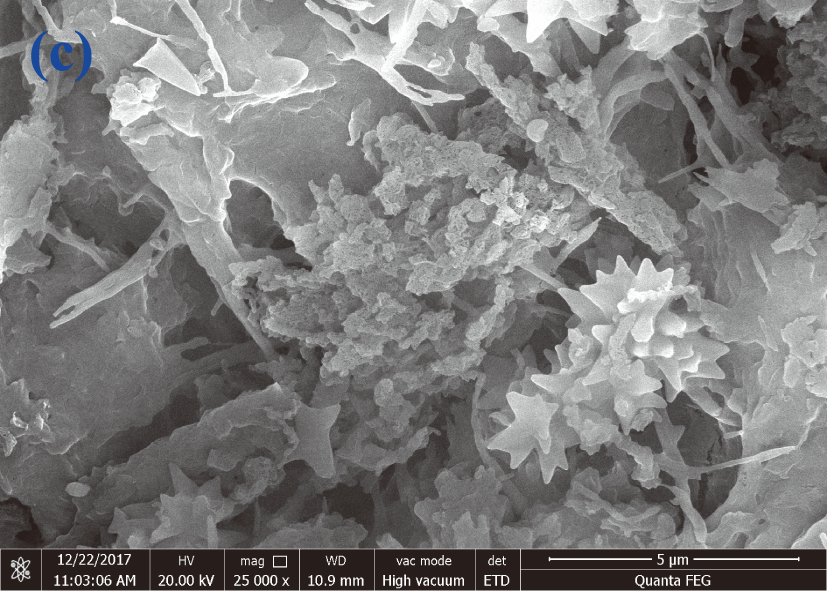

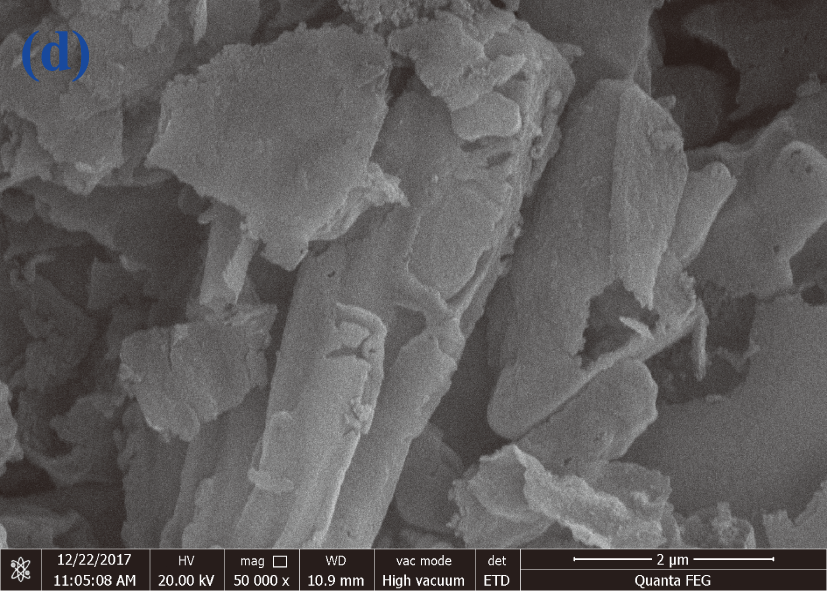


**Fig. S8**. SEM images of CNs under different solvents. (a) H_2_O, (b) CH_3_COOH, (c) TEP, (d) DMF.

**1.2 Simulation methods**

All simulation calculations using the molecular simulation software Materials Studio at T = 353.15 K and P = 1.0 atm, mainly using the Forcite, Amorphous Cell and Reflex modules. Using COMPASS II force field, and the van der Waals interaction force was calculated using the Atom based method. The spline and buffer widths were 1.00 and 0.50 Å, respectively. The Electrostatic force was calculated using the Ewald method with a calculation of 0.00001 kcal/mol and an update of 1.00 Å. Charges used by Forcefield assigned. In all MD simulations, the time steps are set to 1.0 fs. The Smart Minimizer method is used to minimize the energy of 5000 steps, and the annealing calculation is used to find the optimal configuration.

Firstly, the structural monomer of CN was generated through geometry and anneal optimization (Fig. 2(a)). After that, the above structural monomer was used to build crystal and the space groups were P1, P2, P3 and P4 (Fig. 2(b)) along with 2×2×2 supercell (Fig. 2(c)), respectively. Besides, we used the Amorphous Cell to construct an isotactic chain containing a CN monomer and its configuration was optimized. The final ensemble is transformed into P1, P2, P3 and P4 along with 2×2×2 supercell, respectively (Fig. 2(d)). The XRD patterns were obtained through Reflex.


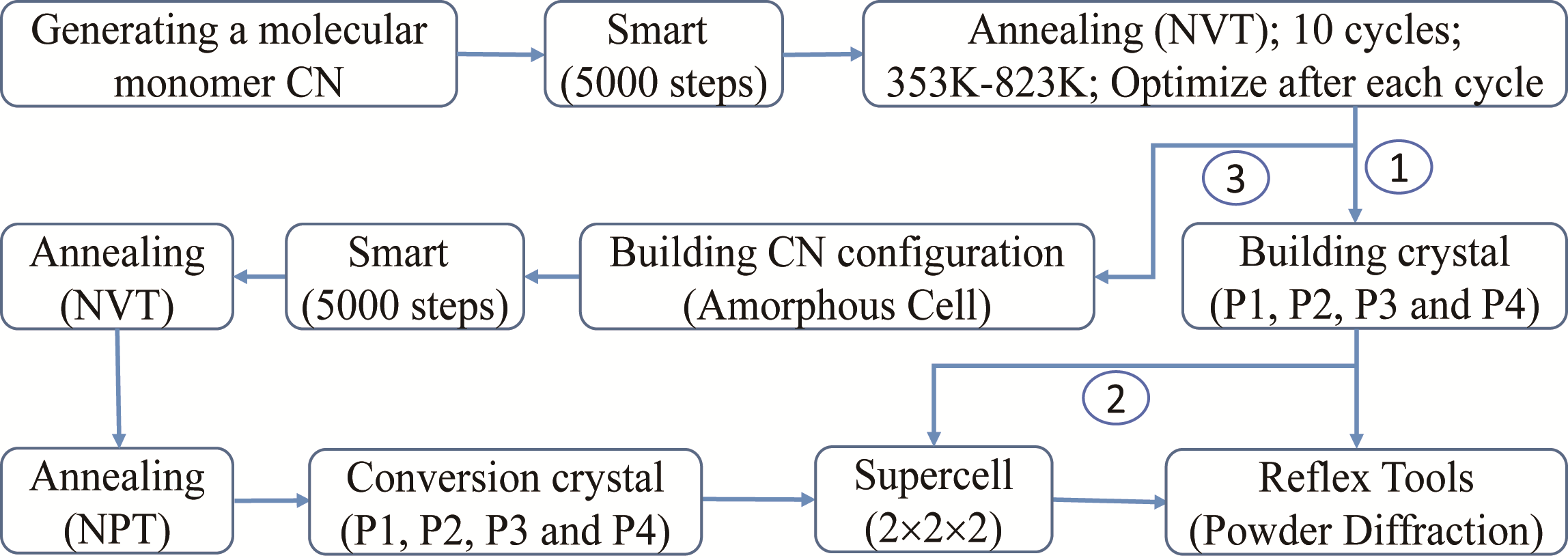


**Fig. S9.** Flow chart of simulation calculation of CN material.

**Reference**

1. Fang LJ, Li YH, Liu PF, Wang DP, Zeng H, Wang X, Yang HG (2017) Facile fabrication of large-aspect-ratio g-C_3_N_4_ nanosheets for enhanced photocatalytic hydrogen evolution. ACS Sustain Chem Eng 5:2039-2043

2. Zhang XS, Hu JY, Jiang H (2014) Facile modification of a graphitic carbon nitride catalyst to improve its photoreactivity under visible light irradiation. Chem Eng J 256:230-237

3. Wang J, Li M, Zhou S, Xue A, Zhang Y, Zhao Y, Zhong J, Zhang Q (2017) Graphitic carbon nitride nanosheets embedded in poly(vinyl alcohol) nanocomposite membranes for ethanol dehydration via pervaporation. Sep Purif Technol 188:24-37
